# Brain imaging and machine learning reveal uncoupled functional network for contextual threat memory in long sepsis

**DOI:** 10.21203/rs.3.rs-4870916/v1

**Published:** 2024-10-15

**Authors:** Patricio T. Huerta, Joshua J. Strohl, Joseph Carrión

**Affiliations:** Feinstein Institutes for Medical Research, Northwell Health; Feinstein Institutes for Medical Research, Northwell Health; Feinstein Institutes for Medical Research, Northwell Health

## Abstract

Positron emission tomography (PET) is a highly sensitive tool for studying physiology and metabolism through positron-emitting radionuclides that label molecular targets in the body with unparalleled specificity, without disturbing their biological function. Here, we introduce a small-animal technique called behavioral task–associated PET (beta-PET) consisting of two scans: the first after a mouse is familiarized with a conditioning chamber, and the second upon recall of contextual threat. Associative threat conditioning occurs between the scans. Beta-PET focuses on brain regions encoding threat memory (e.g., amygdala, prefrontal cortex) and contextual aspects (e.g., hippocampus, subiculum, entorhinal cortex). Our results show that beta-PET identifies a biologically defined functional network encoding contextual threat memory and its uncoupling in a mouse model of long sepsis. Moreover, machine learning algorithms (linear logistic regression) and ordinal trends analysis demonstrate that beta-PET robustly predicts the behavioral defense response and its breakdown during long sepsis.

## INTRODUCTION

Positron emission tomography (PET) is a well-established imaging technique in which compounds labeled with positron-emitting radioisotopes are used as molecular probes to image and measure physiological processes in health and disease^1,2^. For instance, the presence and severity of cancers, cardiovascular disease, and brain disorders are routinely probed with PET scans^1–3^. The commonly used PET radiotracer [^18^F]fluorodeoxyglucose (FDG) allows for quantitative measurements of glucose uptake, given that FDG PET generates detailed patterns of glucose utilization across the whole body. Within the brain, these metabolic patterns are interpreted as nodes of neural activity that make up functional networks^4^. FDG PET has been widely applied to brain diseases in both clinical^5–8^ and preclinical settings^9^. While much has been learned from these studies, using FDG PET in conjunction with behavioral assays remains underutilized. Notably, after an FDG injection, there is a time window when behavioral tasks can be performed so that the FDG signal may reflect the brain functional network associated with the task^10^. Therefore, by combining FDG PET with behavioral tasks, the nodes engaged by the task can be examined in the healthy as well as the diseased brain.

Raw brain PET data are often analyzed with an approach called statistical parametric mapping (SPM), which compares two experimental groups by performing thousands of Student’s t tests on a voxel-by-voxel basis across the brain^11^. SPM produces statistically defined clusters of contiguous voxels, and the brain regions that share a space with the statistically significant voxels are reported. Recently, multivariate methods have been developed for finding networks of correlated activity throughout the brain. One such method, called ordinal trends (ORT) canonical variates analysis, applies supervised principal component (PC) analysis across PET datasets that involve multiple conditions within the same subjects. The PC, or combination of PCs, that leads to the greatest variance within each subject across conditions is identified and its expression is calculated for each subject^4,12,13^.

Associative threat conditioning (formerly referred to as fear conditioning^14^) consists of a behavioral paradigm in which a subject is exposed to a conditioned stimulus, for example a series of tones, which is paired with an unconditioned stimulus, often a mild foot-shock. Acquisition of the aversive memory causes the subject to associate the conditioned stimulus (tone) as well as the context (conditioning chamber) with the unconditioned stimulus (foot-shock)^15,16^. Subsequent presentations of the tone or context elicit a defense response, the acquisition and expression of which engage the amygdala and prefrontal cortex^16–24^, which are considered the neural substrate for threat memory. In addition, the temporal and contextual elements of threat memory are encoded by the hippocampus and associated regions^17,25–29^. These structures, which are critically important in the healthy brain, are highly susceptible to diseases that involve inflammation^9,30^.

Sepsis is the overwhelming response by the body to infection, leading to organ damage^31^. Long-term survivors of sepsis suffer chronic immune dysregulation, brain fog, and cognitive impairment long after the initial insult^32–38^. We have termed this critical but understudied condition ‘long sepsis’ (LS) and have modeled it in preclinical studies^39,40^. While behavioral and anatomical results have indicated that the hippocampus is affected by LS, the brain-wide neural networks involved have not yet been fully identified^39,40^.

This study examines the functional networks of the brain in a mouse model of chronic inflammation resulting from LS. We introduce a technique called behavioral task–associated PET (beta-PET) consisting of two FDG PET scans taken across different phases of the behavioral task. The first scan is done immediately after the animal is familiarized to the behavioral apparatus (e.g., the conditioning chamber) and it represents the baseline condition. The second scan occurs after the animal undergoes the recall of contextual threat. The mouse is subjected to associative threat conditioning in the interval between the first and second scans. An important feature of beta-PET is the focused scrutiny of brain regions that encode threat memory (e.g., amygdala and prefrontal cortex) as well as its contextual elements (e.g. hippocampus and associated areas), such that the FDG PET signals from the first scan are subtracted from those of the second scan. The differential signal from beta-PET isolates the PET readout associated to the behavioral task and reduces the influence of metabolic activity that is not relevant to the task in question. Our results show that beta-PET identifies a biologically defined functional network encoding contextual threat memory. Moreover, using machine learning tools and ORT analysis, we demonstrate that the brain network based on beta-PET robustly predicts the behavioral defense response and its breakdown in a mouse model of LS.

## RESULTS

**Figure 1:**
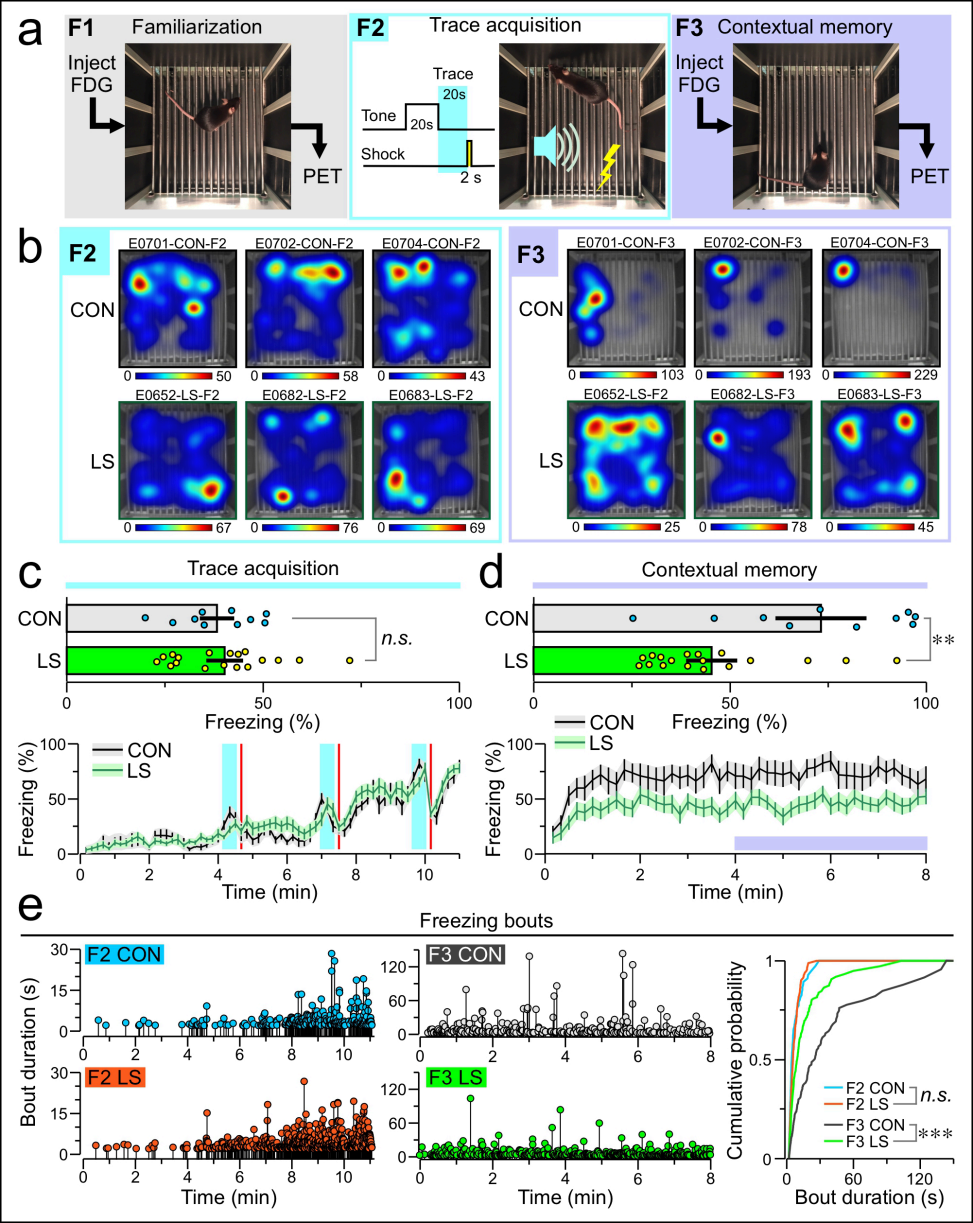
Associative threat conditioning combined with FDG PET. (**a)** Experimental design over the course of 3 days. On day 1, mice are injected with FDG, placed into the conditioning chamber for familiarization (F1), and subjected to a PET scan. On day 2, mice are exposed to trace acquisition (F2), consisting of a 20-s tone, a 20-s trace interval, and a 2-s foot-shock, repeated 3x. On day 3, mice are injected with FDG, returned to the conditioning chamber to test contextual memory (F3), and subjected to a second PET scan. (**b)** Representative heatmaps depicting mouse occupancy during F2 (*left*) and F3 (*right*) for 3 control mice (CON, *top*) and 3 long sepsis mice (LS, bottom). Heat scales are in seconds. **(c)** *Top*, bar graph showing F2 freezing (mean ± SEM) during the tone presentation and the trace interval (each dot represents a mouse) for CON and LS mice; *n.s*., non-significant, *P* = 0.67 (t test). *Bottom*, plot showing F2 freezing (mean ± SEM) time-series using 10-s bins. **(d)** *Top*, bar graph showing F3 freezing (mean ± SEM) for last 4 min of contextual memory test (each dot represents a mouse) for CON and LS mice; **, *P* = 6.85 × 10^−3^ (t test). *Bottom*, plot showing F3 freezing (mean ± SEM) time-series using 10-s bins. **(e)**. *Left*, lollipop plots showing the start time and duration of each individual freezing bout during F2 and F3. *Right*, cumulative probability curve for bout duration in each group across F2 and F3; ***, *P* = 4.49 × 10^−4^ (Kolmogorov-Smirnov test).

We sought to understand how chronic inflammation affects the brain using a mouse model of long sepsis. Experimental mice (termed LS henceforth) were subjected to cecal ligation and puncture (CLP), whereas control mice (termed CON henceforth) underwent sham surgery. All animals were given a period of approximately 6 weeks to recover from the surgery. Associative threat conditioning took place over the course of 3 days (Fig. 1a); the first and third days included FDG PET scans. On day 1, mice were injected with FDG, left undisturbed for 20 min (allowing time for tracer uptake), placed into the conditioning chamber for a familiarization session (F1), anesthetized and subjected to the first scan. On day 2, mice underwent trace acquisition (F2), which consisted of 3 repetitions of a tone (80 dB, 5 kHz, 20-s long), trace interval (20 s) and a mild foot-shock (1 mA, 2-s long). On day 3, mice were tested for contextual memory (F3), with FDG injections and PET scans occurring exactly as on day 1. Assessment of the defense response reveals that both CON (*n* = 10) and LS (*n* = 18) groups have similar rates of freezing during F2 (Fig. 1b, c) (mean ± SEM, CON = 38.27 ± 3.23%, LS = 40.22 ± 3.12%, *T* = 0.44, *P* = 0.67, t test). However, LS mice freeze significantly less compared to CON mice during F3 (Fig. 1b, d) (CON = 73.21 ± 7.73%, LS = 45.37 ± 4.34%, *T* = 3.14, *P* = 6.85 × 10^−3^, t test). We also find that the CON group exhibits significantly longer freezing bouts during F3 (Fig. 1e, D = 0.145, *P* = 4.49 × 10^−4^, Kolmogorov-Smirnov test). Of the 28 mice scanned, there were 4 animals for which the scan failed due to technical issues, yielding 9 CON and 15 LS scans.

**Figure 2:**
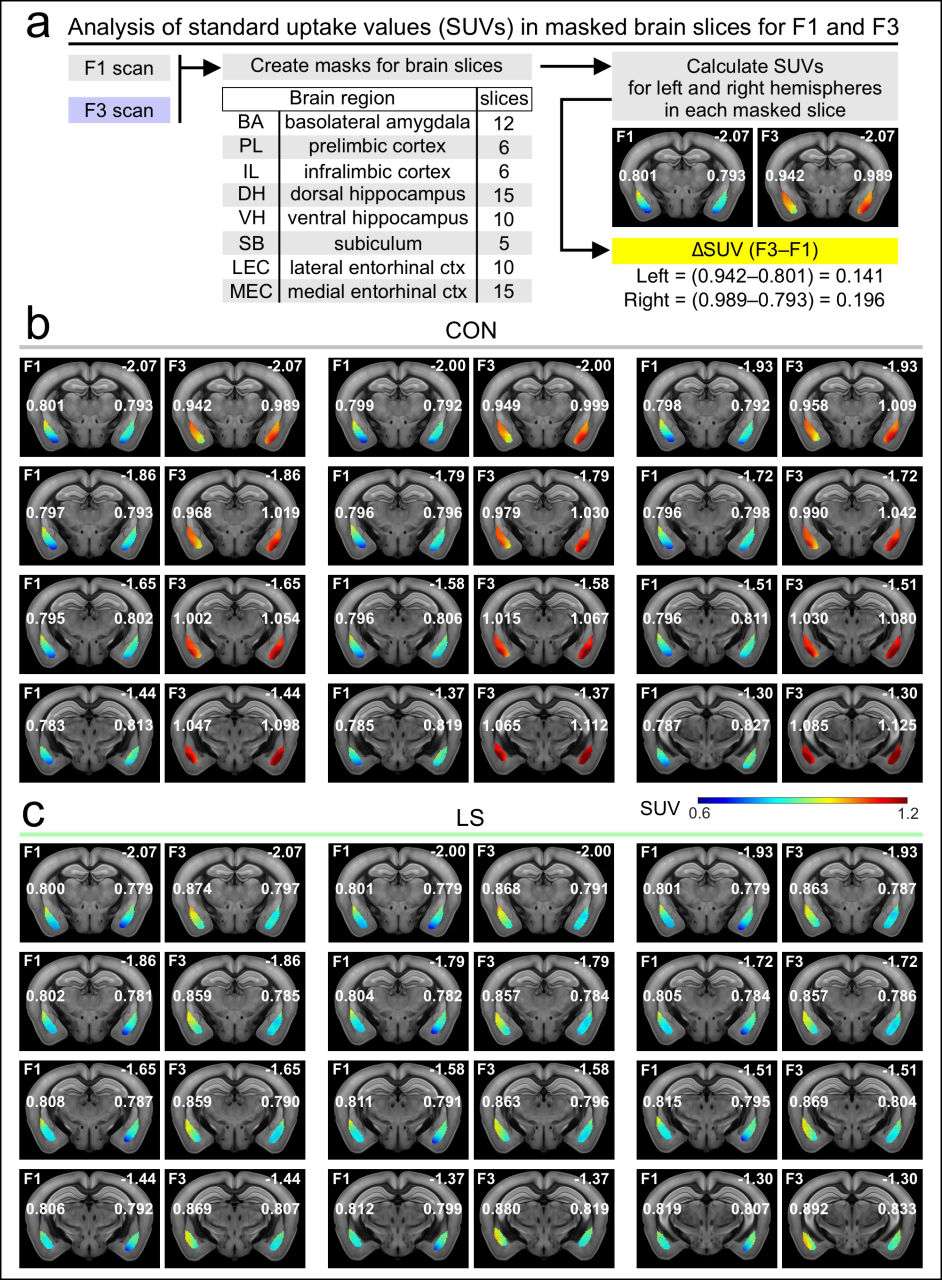
Demonstration of the beta-PET approach. **(a)** Workflow for beta-PET. Masks are drawn into coronal brain slices [ref. 49, 50] and the number of slices (for the 8 brain regions) is indicated. Standard uptake values (SUVs) are calculated for the masks, and the values for F1 are subtracted from the values for F3 to obtain DSUV(F3–F1). **(b)** Representative scans from a CON mouse showing the 12 slices used for BA. The F1 and F3 scans are shown side by side for each slice, with the heatmap scaled to the SUV. The value of each hemisphere (within each slice) is indicated. The number at the top right of each slice represents the distance from bregma (in mm). **(c)** Representative scans for the BA analysis in a LS mouse, similar as in (b).

**Figure 3:**
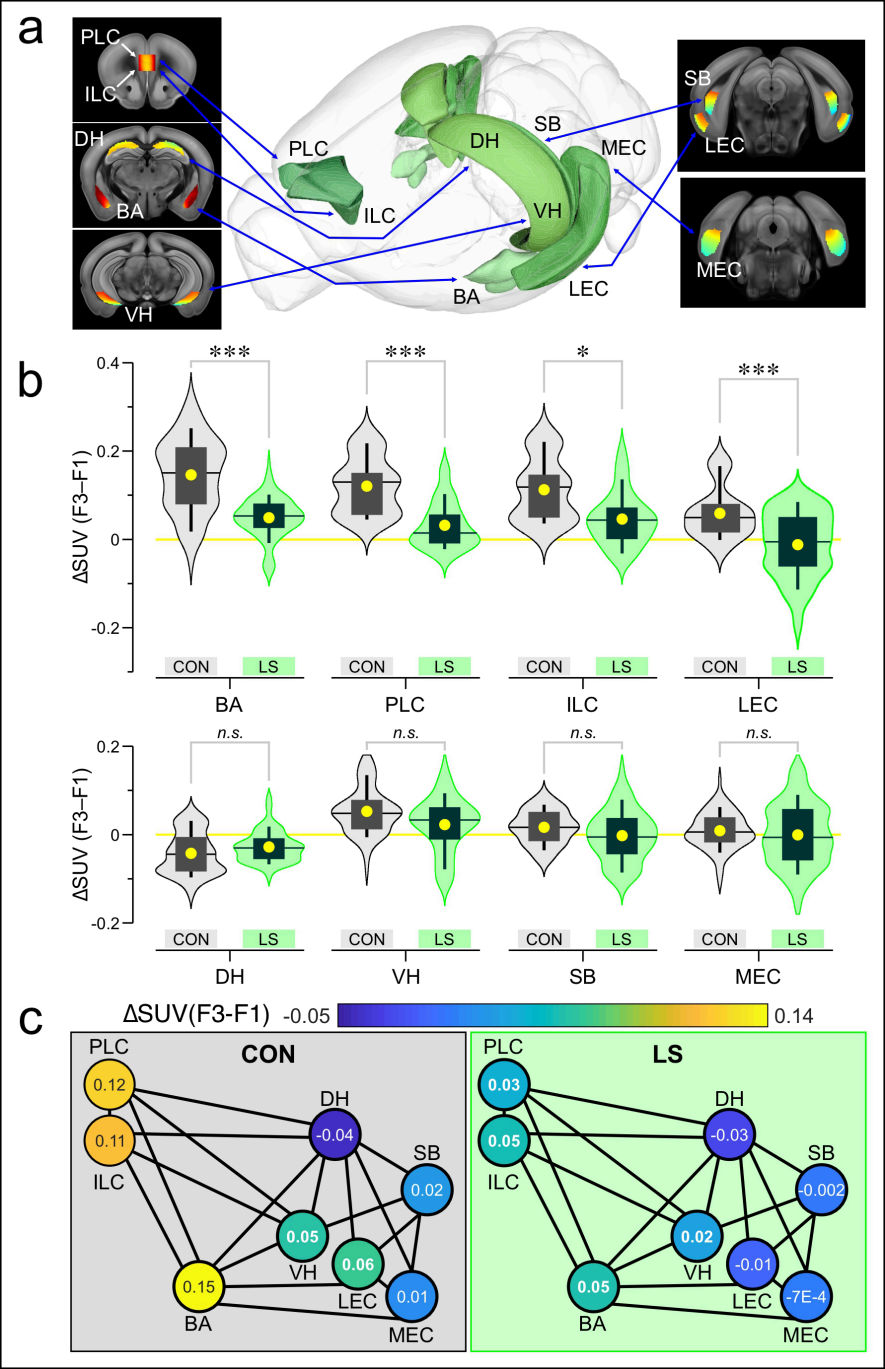
Beta-PET applied to the functional network encoding threat memory. **(a)** Diagram showing 3D renderings of the brain regions under study [ref. 51, 52] alongside coronal sections showing SUVs averaged across all CON mice during F3. Abbreviations: BA, basolateral amygdala; DH, dorsal hippocampus; ILC, infralimbic cortex; LEC, lateral entorhinal cortex; MEC, medial entorhinal cortex; PLC, prelimbic cortex; SB, subiculum; VH, ventral hippocampus. **(b)** Violin plots show kerned smooth distribution of DSUV data where each point is taken from a hemisphere for each slice. Box-and-whisker plots show mean (circle), median (line), inter-quartile range (box), and 10–90 distribution (whiskers). *Top*, brain regions with significant differences: BA, ***, *P* = 2.57 × 10^−5^; PLC, ***, *P* = 1.3 × 10^−4^; ILC, *, *P* = 0.014; LEC, ***, *P* = 2.8 × 10^−4^; statistics with linear mixed model. *Bottom*, brain regions with non-significant (*n.s*.) differences: DH, *P* = 0.27; VH, *P* = 0.19; SB, *P* = 0.34, MEC, *P* = 0.68. **(c)** Network map showing the DSUV for each brain region averaged across all mice from CON (*left*) and LS (*right*) groups; each node represents a brain region. The heatmap for each node is scaled to the DSUVs, with the average DSUV indicated by the number shown within each node.

To elucidate how the brain networks that encode aversive memory (including its contextual aspects) fail during LS, we isolated the FDG PET signals associated with the behavioral task. There is strong agreement on the neural substrates for contextual threat memory^18–21,25,41–48^, which allowed us to make informed decisions about which brain regions to select for beta-PET. Using coronal sections, we analyzed each slice by drawing a mask around a region-of-interest, independently for the left and right hemispheres, except for the prefrontal cortex regions which were analyzed as a single object for each slice^49,50^ (Fig. 2a). We then calculated the standard uptake value (SUV) for each masked slice and subtracted the F1 SUVs from the F3 SUVs to obtain ΔSUVs (Fig. 2a). Given the nested nature of analyzing multiple slices and hemispheres for each mouse, we used general linear–mixed–model (LMM) statistics to account for the multiple values arising from each mouse. Using this differential approach in CON (Fig. 2b) and LS (Fig. 2c) groups, we examined the basolateral amygdala (BA), prelimbic cortex (PLC), infralimbic cortex (ILC), dorsal hippocampus (DH), ventral hippocampus (VH), subiculum (SB), lateral entorhinal cortex (LEC), and medial entorhinal cortex (MEC)^51,52^ (Fig. 3a, **Supplementary Data 1**). We chose BA, PLC, and ILC because these regions are the core neural nodes that encode threat conditioning^18–21,25,41–48^. As our study focused on the contextual memory association in threat conditioning, we chose regions that encode spatial context, such as DH, VH, MEC, LEC, and SB^17,25–27,53–60^. Figure 3b shows significantly abrogated responses in LS mice (*n* = 15), when compared to CON mice (*n* = 9) for BA, PLC, ILC and LEC (BA: CON = 0.15 ± 5.7 × 10^−3^ ΔSUV, LS = 0.049 ± 2.5 × 10^−3^, *F* = 18, *P* = 2.57 × 10^−5^; PLC: CON = 0.12 ± 8.5 × 10^−3^, LS = 0.032 ± 5.5 × 10^−3^, *F* = 15.55, *P* = 1.3 × 10^−4^; ILC: CON = 0.11 ± 9.5 × 10^−3^, LS = 0.046 ± 6.2 × 10^−3^, *F* = 6.21, *P* = 0.014; LEC: CON = 0.059 ± 4.3 × 10^−3^, LS = −0.012 ± 4.3 × 10^−3^, *F* = 13.4, *P* = 2.8 × 10^−4^; statistics with LMM). Figure 3b also shows no significant differences between groups for DH, VH, SB and MEC (DH: CON = −0.043 ± 2.9 × 10^−3^ ΔSUV, LS = −0.028 ± 1.7 × 10^−3^, *F* = 1.21, *P* = 0.27; VH: CON = 0.052 ± 3.9 × 10^−3^, LS = 0.023 ± 3.6 × 10^−3^, *F* = 1.72, *P* = 0.19; SB: CON = 0.016 ± 4.0 × 10^−3^, LS = −2.4 × 10^−3^ ± 4.8 × 10^−3^, *F* = 0.93, *P* = 0.34; MEC: CON = 8.6 × 10^−3^ ± 2.6 × 10^−3^, LS = −7.5 × 10^−4^ ± 3.3 × 10^−3^, *F* = 0.18, *P* = 0.68; LMM). When the mean ΔSUVs for each group are shown in a network map (Fig. 3c), the functional responses can be clearly visualized in CON mice (enhanced glucose uptake in BA, PLC, and ILC) as well as their striking absence in the LS group.

**Figure 4:**
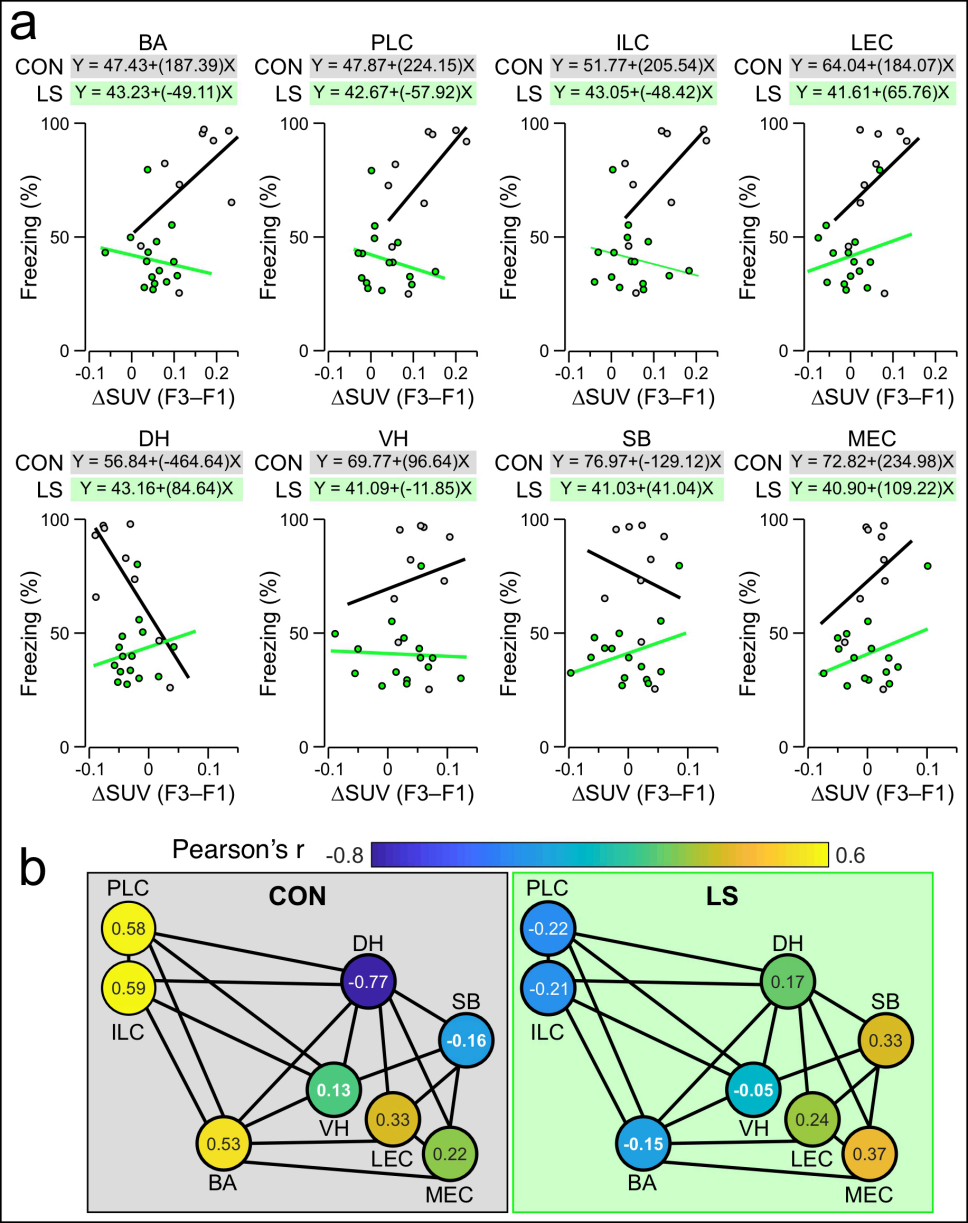
Correlations between beta-PET and behavioral readouts. **(a)** Scatter plots showing correlation of the DSUV for each brain region against % freezing for each mouse (each dot represents a mouse). Grey line represents linear fit for CON mice, green line represents linear fit for LS mice. The equation describing the linear fit for each group is shown above each plot. Abbreviations same as in Fig. 3. **(b)** Network map showing the Pearson’s r values describing the correlation of DSUVs for each brain region with behavior, averaged across all mice from each group (each node represents a brain region). *Right*, average Pearson’s *r*values for CON mice. *Left*, average Pearson’s r values for LS mice. The heatmap for each node is scaled to the Pearson’s *r* values, with the average Pearson’s *r* indicated by the number shown within each node.

We next examined the correlations between ΔSUVs for each mouse and the behavioral readout (% freezing in F3) for each brain region under analysis. We fitted the CON and LS datasets with linear regressions and calculated the Pearson’s correlation coefficient (*r*) for each curve (Fig. 4a). CON mice have the strongest positive correlations between ΔSUV and freezing in ILC (*r* = 0.59), PLC (*r* = 0.58), and BA (*r* = 0.53) as well as a strong negative correlation in DH (*r* = −0.77). Notably, LS mice exhibit a contrasting pattern of correlations (when compared to CON mice) with negative correlations in ILC (*r* = −0.21), PLC (*r* = −0.22), and BA (*r* = −0.15) and a positive correlation in DH (*r* = 0.17). Surprisingly, of all the brain areas examined, LS mice have the strongest positive correlation in MEC (*r* = 0.37), although this correlation is of a lower magnitude than those found in the key areas of CON mice. When the Pearson’s *r* values for each group are shown in a network map (Fig. 4b), the correlated responses can be clearly visualized in CON mice (elevated correlations in BA, PLC, and ILC) as well as the negative values in the LS group.

**Figure 5:**
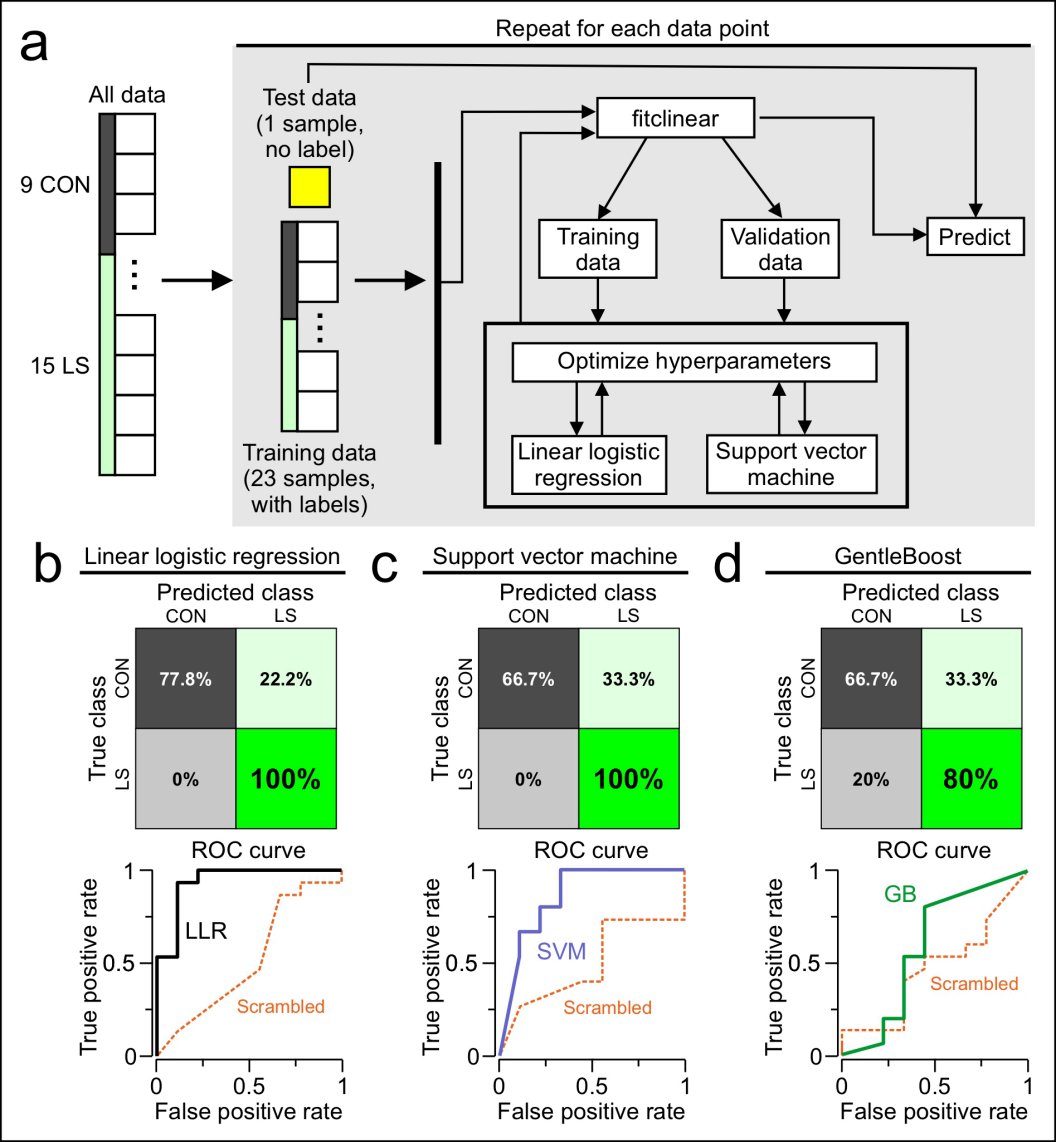
Machine learning predicts class based on beta-PET. **(a)** Diagram illustrating the implementation of leave-one-out cross validation. The dataset consists of 8 features and a class label for each mouse. The features are the beta-PET DSUVs corresponding to each brain region (regions depicted in Fig. 3), and the class label is either CON or LS. For each iteration of the cross-validation loop, one datapoint (with no class label) is chosen as the test data. The test data is excluded from training the model. The remaining labeled datapoints are used as training and validation data, and the *fitclinear* function is used to train the model and tune the hyperparameters. The trained model is used to predict the class of the test data. This loop is repeated with a different mouse assigned to the test data for each iteration, until all of the mice have been classified by the algorithm. The same leave-one-out cross validation approach is also implemented using a scalable vector machine (using the *fitcsvm*function), and GentleBoost (using the *fitcensemble* function). **(b)** *Top*, confusion matrix for the linear logistic regression. The predicted class is shown on the top and the true class is shown on the left. Treating CON as the negative class, and LS as the positive class, the true negative rate is shown in the upper left box (77.8%), the false positive rate is shown in the upper right box (22.2%), the false negative rate is shown in the lower left box (0%), and the true positive rate is shown in the lower right box (100%). *Bottom*, receiver operating characteristic (ROC) curve for the linear logistic regression. The performance of the algorithm is shown in the solid line, while the dashed line shows the performance of the algorithm when the classes and features are scrambled. **(c)** Confusion matrix and ROC curve for support vector machine, similar as in (b). **(d)** Confusion matrix and ROC curve for GentleBoost, similar as in (b).

To evaluate the efficacy of our results in identifying the chronic inflammation condition, we decided to apply a machine learning approach. Our goal was to predict the class of each mouse as either CON or LS based solely on PET data. We tested three different machine learning classification algorithms, using the ΔSUVs (of each region-of-interest) as our input features. The algorithms included linear–logistic–regression (LLR), support–vector–machine (SVM), and an ensemble-tree based method using GentleBoost. We performed these operations in MATLAB using the functions *fitclinear* (for LLR), *fitscsvm* (for SVM), and *fitcensemble* (for GentleBoost). We implemented a leave-one-out cross validation approach to account for the relatively small sample sizes of our datasets. This approach was done using a loop where one mouse was taken out for each iteration to be used as the test data. The classification model was trained on the remaining mice, with the MATLAB functions automatically splitting the data into training and validation datasets. The *fitclinear* function selected between LLR and SVM classifiers as a hyperparameter and chose an LLR, based on the training and validation data (Fig. 5a). Nevertheless, we show the results from *fitcsvm* and *fitcensemble* for comparison, although these models did not perform as well as the LLR. Treating CON as the negative class, and LS as the positive class, we found that the LLR model performed exceptionally well, giving a true negative rate of 77.8% a true positive rate of 100%. We created ROC curves for each classifier and scrambled the labels for training the models (to determine if our models fared better with the real data than the scrambled data). For the LLR, the curve with the real data showed much better classification than that for the scrambled data, which resembled that of a random classifier (Fig. 5b). We found the SVM model did not perform as well as the LLR, with a true negative rate of 66.7% and a true positive rate of 100%. The ROC curve for the real data showed better classification than for the scrambled data, but this was not as impressive as the LLR (Fig. 5c). The Gentleboost model performed the worst with a true negative rate of 66.7% and a true positive rate of 80%. The ROC curve for this dataset indicated a performance only slightly better than that of the scrambled data, indicating that this model would be an inappropriate choice for our data (Fig. 5d).

**Figure 6:**
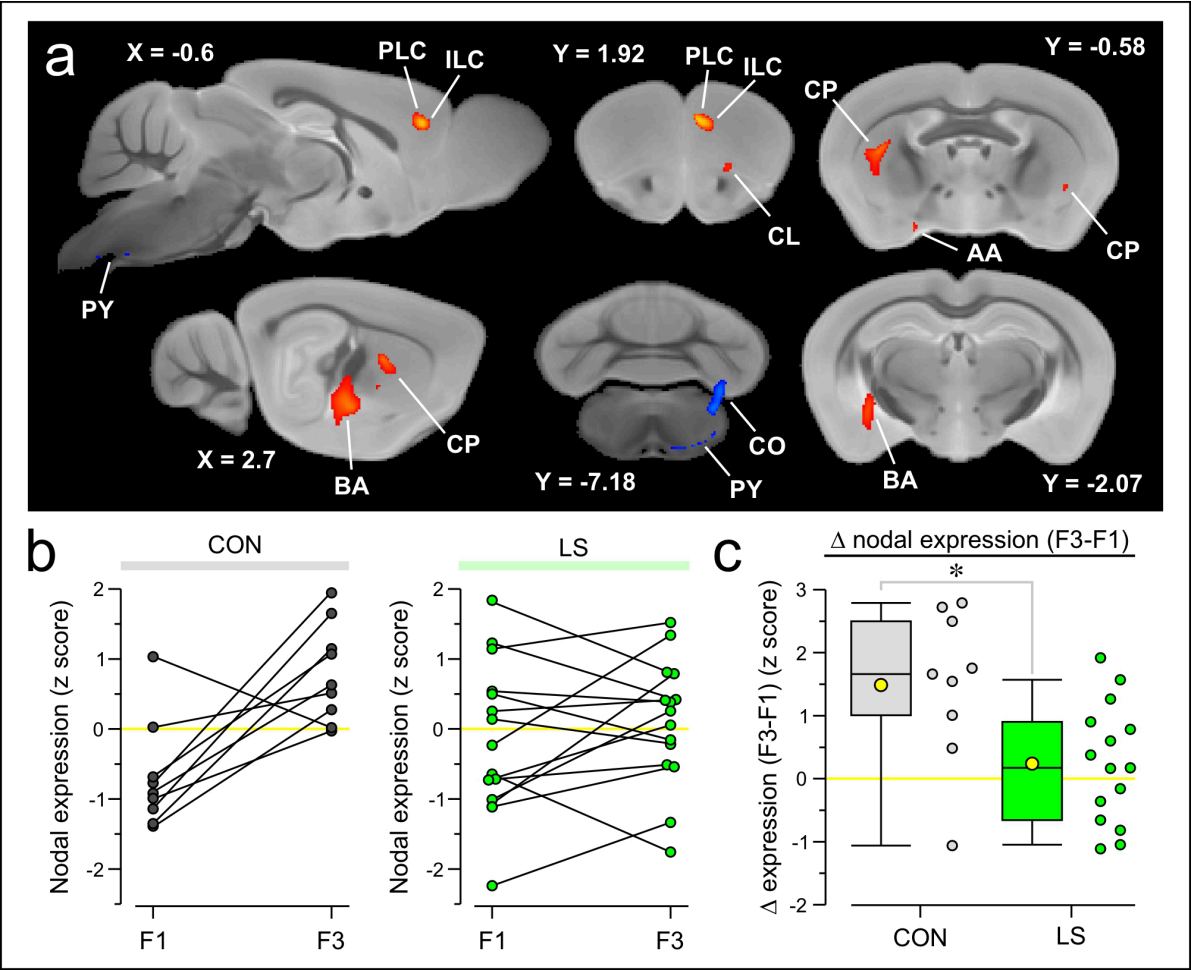
Ordinal trends analysis confirms behavior-associated nodes. **(a)** Statistically significant voxels displayed over mouse brain MRI template. Ordinal trends (ORT) analysis is performed on CON mice using scans from F1 and F3, and the PC with the greatest variance across scans is identified. The principal component (PC) is bootstrapped 500 times, and the statistically significant voxels are plotted over cross-sections of a mouse brain MRI template. Voxels which increase from F1 to F3 are shown in red, while voxels that decrease from F1 to F3 are shown in blue. The distance from midline (in mm) is shown next to each sagittal slice (e.g., X = −0.6), and the distance from bregma (in mm) is shown for each coronal slice (e.g., Y = 1.92). Regions identified from the significant voxels include the prelimbic cortex (PLC), infralimbic cortex (ILC), basolateral amygdala (BA), claustrum (CL), caudate putamen (CP), anterior amygdala area (AA), copula pyramidis (CO), and the pyramidal tract (PY). **(b)** Line series plots showing the z-scored nodal expression for F1 and F3 of PC indicated in (a). Each pair of connected dots represents 1 mouse. *Left*, CON mice, *right*, LS mice. **(c)**Box-and-whisker plot of the change in nodal expression of the PC indicated in (a) from F1 to F3 (each dot represents a mouse). Box-and-whisker plot shows mean (square), median (line), inter-quartile range (box), and 10–90 distribution (whiskers); *, *P* = 0.02, t test.

Given the strength of our results using the beta-PET approach, we sought to validate it against an accepted multivariate approach for analyzing PET data. Our dataset consisted of two sequential conditions for each mouse, making ORT analysis an appropriate choice, which we applied on the scans of CON mice, using F1 as the first condition and F3 as the second condition. This analysis generated PCs, which were assessed on their own, and in linear combinations with each other using the Akaike information criteria. Using this approach, it was determined that one PC (PC3) had the best fit. This PC was found to exhibit a statistically significant ordinal trend using the permutation test (*P* = 0.018). The voxel weights for the ORT were evaluated using bootstrap resampling to ensure reliability, and the statistically significant voxel weights (*P* < 0.05, one tailed, 500 iterations) were plotted over a magnetic resonance imaging (MRI) template that was aligned with the PET scans. This approach found significant voxel weights in PLC, ILC, and BA, thus confirming our beta-PET approach. Interestingly, ORT analysis also identified other regions including the caudate putamen (CP), claustrum (CL), anterior amygdala area (AA), copula pyramidis (CO) which is part of the cerebellum, and pyramidal tract (PY) within the brainstem (Fig. 6a). We plotted the nodal expression of the identified PC for CON and LS mice from F1 to F3 and found that 88.9% of CON mice had an increase in nodal expression, compared to only 60% of LS mice (Fig. 6b). We then subtracted the change in nodal expression from F1 to F3, which revealed a significantly lower expression in LS mice when compared to CON mice (Fig. 6c, CON = 1.49 ± 0.41 z-score, LS = 0.24 ± 0.94, *T* = 2.62, *P* = 0.02, t test).

## DISCUSSION

We have studied the brain metabolic patterns that are engaged in response to the contextual memory of an aversive event. Through the use of several analytical methods, we have shown that these metabolic patterns fail to activate following chronic inflammation. We have introduced the beta-PET technique (Fig. 2, 3), which involves performing FDG PET scans in two phases of a behavioral task, and then using a differential region-of-interest analysis to measure the response in the chosen brain areas. Notably, the beta-PET results correlate with the behavioral readout (% freezing in F3) (Fig. 4) and are highly accurate in differentiating between CON and LS mice with machine learning classification algorithms (Fig. 5). Furthermore, this approach enabled us to generate a map across the multiple brain areas we examined, providing a clear visualization of the way in which LS alters the brain network responsible for encoding contextual threat memory (Fig. 3c). We have confirmed our results using ORT analysis (Fig. 6), a multivariate approach which identified brain areas overlapping with those examined with beta-PET. While the brain regions identified by ORT analysis agreed with those used in beta-PET, ORT analysis also highlighted a few regions that are unrelated to contextual threat memory. These areas include parts of the cerebellum, striatum, and brainstem. We postulate that the metabolic changes detected in these regions are a consequence of the mouse’s reduced motility during F3 rather than a cognitive process associated with contextual memory. Notably, the beta-PET approach allows us to focus on the brain regions related to the task under investigation. Thus, we think this overall approach is well suited to study a wide variety of behavioral tasks and brain disorders.

In our study, the brain areas chosen for beta-PET are essential components of the brain’s threat network, particularly the prefrontal cortex and amygdala^17,20,21,25,44^. Indeed, the change in metabolic activity in these regions is strongly correlated with the level of freezing during the contextual memory test in CON mice. Notably, not only is the change in metabolic activity smaller in LS mice, but there is also uncoupling in the correlation between metabolism and behavior. Previous studies using the LS model had focused on the hippocampus as the neural substrate for cognitive impairment^39,40^, but the present results suggest that there are broader neural abnormalities which extend to the prefrontal cortex and amygdala.

Despite the sparsity of human studies in people who have survived sepsis, cognitive impairment and brain dysfunction seem to be pervasive conditions that occur in practically all sepsis survivors^33,36,61–65^. We believe our preclinical findings provide a roadmap for clinical studies on long-term sepsis survivors. Brain imaging studies in conjunction with threat conditioning have been conducted in humans, and the brain areas that are engaged by threat conditioning are largely analogous to other mammalian species^66^. Therefore, by combining human neuroimaging with threat conditioning in sepsis survivors, we might be able to learn about this critically understudied condition in patients. Crucially, our work demonstrates that mice can be classified using machine learning algorithms (as either CON or LS) using only data from the PET signals. This provides additional translational impact, as PET can be used in clinical settings, and the examination of brain functional networks might provide a powerful biomarker for brain dysfunction in sepsis patients following their recovery from the acute phase of this condition.

A limitation of this study is the use of the CLP model, which has recently come under heavy scrutiny as being a poor model of sepsis^67,68^. While we acknowledge the criticisms of this model, we would like to point out three considerations. First, the vast majority of studies that have used the CLP model have examined the first 24–72 hours after the procedure, while our model investigates survivors several weeks after recovery; indeed, the long-term sequalae of sepsis remain largely understudied in preclinical models as well as in human patients. Second, most studies using CLP have focused heavily on specific immunological dysregulations triggered by the septic shock, often offering the modification of a single immune factor as a potential therapeutic strategy. Unsurprisingly, this reductionist approach has led to several failed clinical trials in humans. In contrast, the LS model we present applies a broader systems neuroscience perspective, which is removed from the initial immunological trigger(s). Third, the Sepsis-3 definition states that sepsis is a life-threatening organ dysfunction caused by an abnormal host response to infection^31^. We think the brain dysfunction we have identified in our LS model fits smoothly into the Sepsis-3 definition and offers important translational insights. Our murine findings point to the brain’s network encoding contextual threat memory as a biomarker of organ dysfunction in human sepsis survivors.

## METHODS

### Ethics statement:

Animal experiments were performed in accordance with the National Institutes of Health (NIH) Guidelines under protocols approved by the Feinstein Institutes for Medical Research Institutional Animal Care and Use Committee (IACUC). Our Animal Research Program is registered with the Department of Health and Human Services (DHHS), Office of Laboratory Animal Welfare (OLAW), U.S. Department of Agriculture (USDA #21R0107), Public Health Service (PHS #A3168–01) and New York State Department of Health (NYSDOH #A-060). Efforts were performed to minimize the number of animals used and their suffering. All procedures involving experimental animals were performed in accordance with ARRIVE guidelines, reviewed and approved by the Feinstein’s IACUC, protocol code #2023–003.

### Mice:

C57BL/6 mice were purchased from the Jackson Laboratory (Bar Harbor, ME) and maintained on a 12-h reverse circadian cycle (dark 9:00–21:00, light 21:00–9:00) with ad libitum access to water and chow. After arrival, mice were left undisturbed for one week prior to the start of any handling or experiments to allow for acclimation. Mice were initially housed 5 per cage. All experiments were performed during the dark phase of the circadian cycle.

### Cecal ligation and puncture procedure:

At 8 weeks of age, mice underwent either CLP or sham surgery. Mice were anaesthetized using isoflurane (1.5–2.5%) and the surgical site was shaved and wiped clean with betadine solution and isopropyl alcohol. The peritoneal cavity was opened and the cecum ligated below the ileocecal valve using 4–0 silk suture. The cecum was then punctured using a 22G needle and ~1mm of stool was extruded. The cecum was placed back into the mouse, and the incision site was closed with 6–0 ethilon silk (Ethicon, NJ) and wound clips (Stoelting, IL). For the sham operation, mice were opened, the cecum was exposed, and the incision site was closed with no ligation or puncture made to the cecum. All mice received 0.5 mL saline resuscitation, a single dose of buprenorphine (Buprenex, 0.03 mg per kg), and a single dose of antibiotics (primaxin, Merck, Kenilworth, NJ, 0.5 mg per mouse, in 0.2 mL sterile saline) immediately following the surgery via intraperitoneal injection.

### Mouse handling:

Mice were handled for 15 min per day for 3 days prior to the start of behavioral tasks, in a setting with high light. Importantly, handling occurred in a different room from where behavioral experiments were carried out.

### Associative threat conditioning:

We have previously published a variant of these procedures^30^. Videos from all behavioral experiments were recorded using an overhead camera. The videos were saved, tracked, and analyzed using EthoVision XT 14 (Noldus, VA). The same software was used to control the administration of the tones and foot-shocks. Threat conditioning was performed over the course of three days. On day 1, mice were placed in the conditioning chamber (18 cm × 18 cm × 30 cm) for a familiarization session of 10 min and returned to their home cages. On day 2, mice were placed back into the chamber and, over the course of 11 min, were subjected to three 20-s long tones (80 dB, 5 kHz), each of which was followed by a 20-s trace period and a foot-shock (1 mA, 2-s long). Contextual memory was tested on day 3 by returning mice to the conditioning chamber for 8 min with no tones or foot-shocks.

### Freezing analysis:

The activity analysis feature in EthoVision tracked the number of pixels that changed from frame to frame and determined the amount of movement or freezing by calculating how many frames showed pixel changes. These activity scores for every video frame were imported into a custom script in MATLAB (MathWorks, Inc., Natick, MA) which allowed the user to set a threshold for what was considered activity compared to inactivity. The percent freezing was then calculated on a 10-s interval basis, which reflected the percent of frames with inactivity over this interval. The bout analysis was performed with a custom MATLAB script that used the unbinned activity data and calculated the start time and duration of each freezing bout (periods of 2 s or longer were considered as bouts).

### FDG PET:

Mice were injected intraperitoneally with FDG (0.5–1.0 mCi), and tracer uptake was allowed for 20 min prior to placement into the conditioning chamber during the familiarization and contextual memory sessions. The time between FDG injection and placement into the chamber was chosen to allow for maximum uptake during the behavioral task. After F1 or F3, mice were anesthetized with 2.5% isoflurane and placed onto the Siemens Inveon PET scanner (Siemens SG, Munich, Germany) where anesthesia was maintained using 2% isoflurane. A 10-min emission scan followed by an 8-min transmission scan were obtained and reconstructed. The final matrix had a size of 128 × 128 × 159 mm with a pixel size of 0.78 × 0.78 × 0.8 mm.

### PET scan preprocessing:

All acquired images were preprocessed using PMOD 3.3 (PMOD Technologies Ltd., Zurich, Switzerland) prior to further analysis. First, the brain was isolated from the rest of the body by cropping acquired images to a box of size 4.9 × 20.7 × 11.9 mm and aligned to an anatomical template which was generated from MRI scans of male C57BL/6 mice. All non-brain metabolic regions were removed by loading the scans together with a brain mask file created from the brain template and using the PMOD manual co-registration functions to eliminate all pixel values outside the brain area. After PMOD preprocessing, images were analyzed with MATLAB using the SPMMouse package within SPM 5.0 (Wellcome Department of Imaging Neuroscience, Institute of Neurology, London, UK). All images were registered using the *realign* and *reslice* function twice, first aligning to a template image and then aligning to the mean of all images, and then smoothing was performed. The resulting images were used for further analysis.

### Region-of-interest analysis:

It was done using MATLAB (version 2024a). We generated templates for all regions-of-interest by taking the MRI templates to which the scans were aligned, and then drawing masks into coronal slices for each region based on an anatomical atlas^69^. The preprocessed and registered images were loaded into MATLAB and normalized to the mean value for each mouse. Then, masks were applied to each scan to obtain SUVs for each hemisphere of each coronal slice that was drawn. Heatmap images were created for the SUVs for each region in MATLAB using the JET colormap and overlaying onto the corresponding coronal slices downloaded from the Allen Brain Map Reference Atlas^49,50^ for visualization purposes. For each mouse, we subtracted the F1 SUVs from the F3 SUVs of each hemisphere within each slice and represented these values as DSUVs.

### Machine learning classifiers:

We used the Statistics and Machine Learning Toolbox in MATLAB (version 2024a). As input data, we used the DSUVs obtained from the region-of-interest analysis. For each iteration of the loop, one mouse was isolated from the rest of the dataset to be used as test data. The remaining mice were then used as the training and validation data to train the model and optimize the hyperparameters. We used the *fitclinear, fitcsvm*, and *fitcensemble* functions to create the model for each loop. We then applied the predict function in MATLAB, using the trained model to predict the class of the test data. This entire process was repeated for each iteration of the leave-one-out cross validation loop.

### ORT analysis:

Preprocessed and registered images from CON mice from F1 and F3 were used as the two conditions for this approach, which was implemented with a custom MATLAB script. The Akaike information criterion for each PC (and combination of PCs) was applied, and the lowest score for this value was used for further analysis. The result was bootstrapped 500 times, and a permutation test with 500 iterations was performed. The bootstrapped voxel weights were shown overlaid onto an MRI template to which the PET scans had been aligned. This was displayed using FSL (FMRIB Software Library version 6.0.1, Analysis Group at the Wellcome Centre for Integrative Neuroimaging, Oxford, UK)^70^. The nodal expression was then evaluated for each mouse, and the difference in expression from F1 to F3 was calculated.

### Statistical analysis:

It was carried out using Origin Pro (version 2024, OriginLab, Northampton, MA) or the MATLAB statistics and Machine Learning toolbox. Nested datasets were analyzed using mixed model ANOVA. Datasets with normal distributions were assessed for statistical significance using Student’s t test, while datasets with non-parametric distributions were tested using Mann-Whitney U test or Kolmogorov-Smirnov test. *P* < 0.05 was used as the cutoff for statistical significance.

## Data Availability

The authors declare that all the datasets supporting the findings of this study are available within the manuscript and its supplementary information files. The MATLAB code used to generate SUVs and perform machine learning classification is available at: https://github.com/HuertaLab/20240730_beta_PET (https://doi.org/10.5281/zenodo.13136023).
